# Supplementary material for: Decoding WW domain tandem-mediated target recognitions in tissue growth and cell polarity
Source: eLife. 2019 Sep 5;8:e49439. doi: 10.7554/eLife.49439 (PMC6744271; doi:10.7554/eLife.49439)
Supplement: Supplementary file 1. [file elife-49439-supp1.docx]

**Supplementary file 1: Statistics of Data Collection and Model Refinement.**

|  | **KIBRA/**  **Lats1-Se-Met** | **KIBRA/**  **LATS1** | **KIBRA/**  **PTPN14** | **KIBRA/**  **AMOT** | **KIBRA/**  **β-DG** | **MAGI2/**  **Dendrin** | **Dendrin-**  **“L”-YAP1** | **YAP1-“L”**  **-Dendrin** |
| --- | --- | --- | --- | --- | --- | --- | --- | --- |
| **Data collection** |  |  |  |  |  |  |  |  |
| Space group | P2_1_2_1_2_1_ | P2_1_2_1_2_1_ | R32 | P2_1_2_1_2 | P321 | P2_1_2_1_2_1_ | P4_1_2_1_2 | P6_3_22 |
| Unit cell (Å) | 44.2,64.8,166.6 | 44.3,62.8,156.8 | 107.6,107.6,107.8 | 65.6,72.3,79.4 | 73.5,73.5,105.3 | 46.9,58.6,86.3 | 77.4,77.4,116.8 | 94.6,94.6,174.4 |
| Resolution (Å) | 50-3.10  (3.15-3.10) * | 50-2.50  (2.54-2.50) | 50-2.40  (2.44-2.40) | 50-2.0  (2.03-2.0) | 50-2.30  (2.34-2.30) | 50-1.65  (1.68-1.65) | 50-2.0  (2.03-2.0) | 30-3.10  (3.15-3.10) |
| Redundancy | 6.7(6.8) | 11.2(9.9) | 9.9(10.3) | 9.9(10.2) | 10.5(10.7) | 6.9(6.8) | 12.8(11.2) | 34.6(35.4) |
| I/*σ*I | 30.0(2.2) | 23.7(2.1) | 18.2(2.7) | 22.4(2.2) | 40.0(2.7) | 44.0(2.5) | 32.8(3.7) | 15.3(2.0) |
| Completeness (%) | 97.5(98.9) | 99.5(91.1) | 100(100) | 99.8(99.9) | 99.7(100) | 99.8(99.9) | 100(100) | 99.9(100) |
| *R*_merge_^a^ | 0.055(0.802) | 0.102(0.982) | 0.124(0.957) | 0.103(0.752) | 0.056(0.886) | 0.047(0.856) | 0.079(0.698) | 0.225(>1) |
| CC_1/2_(highest-resolution shell) | 0.884 | 0.759 | 0.789 | 0.856 | 0.857 | 0.878 | 0.862 | 0.896 |
| No. reflections | 17,894 | 15,085 | 9,363 | 26,123 | 14,385 | 29,302 | 23,948 | 8,870 |
| **Refinement** |  |  |  |  |  |  |  |  |
| Resolution (Å) |  | 50-2.50 | 50-2.40 | 50-2.0 | 50-2.30 | 50-1.65 | 50-2.0 | 50-3.10 |
| *R*_cryst_^b^ / *R*_free_^c^ |  | 0.226/0.274 | 0.190/0.242 | 0.21/0.235 | 0.21/0.253 | 0.206/0.238 | 0.187/0.211 | 0.216/0.243 |
| No. of atoms |  |  |  |  |  |  |  |  |
| Protein/Water |  | 2,190/80 | 904/32 | 2,254/276 | 1,214/75 | 1,693/144 | 1,433/198 | 879/5 |
| B-factors^d^ |  |  |  |  |  |  |  |  |
| Protein/Water |  | 42.1/38.2 | 46.0/47.6 | 41.5/45.3 | 43.8/39.5 | 34.7/39.4 | 32.6/40.2 | 58.3/56.5 |
| R.m.s. deviations |  |  |  |  |  |  |  |  |
| Bond lengths (Å) |  | 0.011 | 0.008 | 0.008 | 0.009 | 0.006 | 0.007 | 0.018 |
| Bond angles (º) |  | 1.261 | 0.984 | 1.14 | 1.101 | 1.003 | 1.103 | 1.265 |
| Ramachandran plot^d^ |  |  |  |  |  |  |  |  |
| Favored/Allowed/Outliers (%) |  | 95.5/4.5/0 | 100/0/0 | 99.3/0.7/0 | 97.9/2.1/0 | 99.4/0.6/0 | 99/1/0 | 94.4/5.6/0 |

Numbers in parentheses represent the value for the highest resolution shell.

a. R_merge_ = Σ |*I_i_* - <*I*>| / Σ*I_i_*, where *I_i_* is the intensity of measured reflection and <*I*> is the mean intensity of all symmetry-related reflections.

b. R_cryst_=Σ||*F*_calc_| – |*F*_obs_||/Σ*F*_obs_, where *F*_obs_ and *F*_calc_ are observed and calculated structure factors.

c. R_free_= Σ_T_||*F*_calc_| – |*F*_obs_||/Σ*F*_obs_, where T is a test data set of about 5% or 10% of the total unique reflections randomly chosen and set aside prior to refinement.

d. B factors and Ramachandran plot statistics are calculated using MolProbity.
