# Supplementary material for: Decoding WW domain tandem-mediated target recognitions in tissue growth and cell polarity
Source: eLife. 2019 Sep 5;8:e49439. doi: 10.7554/eLife.49439 (PMC6744271; doi:10.7554/eLife.49439)
Supplement: Supplementary file 2. [file elife-49439-supp2.docx]

**Supplementary file 2:** **Summary of thermodynamic parameters for the binding of each WW domain or tandem to motifs.**

| **Syringe** | **Cell** | **Kd (nM)** | **N** | **ΔH (kcal/mol)** | **-ΔST (kcal/mol)** |
| --- | --- | --- | --- | --- | --- |
| KIBRA WW12 | PTPN14 PY12 :YAP WW12=1:2 | 8.2±0.6 | 1.1 | -52.7 | 41.7 |
| PTPN14 PY12 | KIBRA WW1 | 32000±7000 | n.r. | n.r. | n.r. |
| PTPN14 PY12 | KIBRA WW2 | n.d. |  |  |  |
| KIBRA WW12 | Dendrin PY23:YAP WW12=1:2 | 2.1±0.2 | 1.1 | -54.9 | 43.1 |
| AMOT PY34 | KIBRA WW12 | 96±6 | 1.1 | -42.4 | 32.8 |
| KIBRA WW12 | AMOT PY12 | 16700±200 | 0.76 | -36.9 | 30.4 |
| KIBRA WW12 | AMOT PY1-4 | n.r. |  |  |  |
| KIBRA WW12 | AMOT PY1 | 49000±7000 | 0.97 | -18.6 | 12.7 |
| KIBRA WW12 | AMOT PY2 | 40000±4000 | n.r. | n.r. | n.r. |
| LATS1 PY23 | KIBRA WW12 | 780±20 | 0.87 | -42.1 | 33.7 |
| LATS1 PY23 Δ553 | KIBRA WW12 | 17±1 | 0.91 | -45.1 | 34.6 |
| KIBRA WW12 | LATS1 PY1-4 | 800±60 | 0.81 | -51.9 | 43.5 |
| KIBRA WW12 | LATS1 PY1-3 | 1000±100 | 1.0 | -50.1 | 42 |
| KIBRA WW12 | LATS1 PY1 | 78000±12000 | n.r. | n.r. | n.r. |
| KIBRA WW12 | Expanded PY12 | 11000±500 | 0.8 | -48.5 | 41.7 |
| PTPN14 PY12 | YAP WW12 | 11600±800 | 0.79 | -27.6 | 20.8 |
| YAP WW12 | Dendrin PY23 | 400±10 | 0.83 | -36.6 | 27.8 |
| YAP WW1 | Dendrin PY23 | 16000±2000 | 1.1 | -24.2 | 17.7 |
| YAP WW2 | Dendrin PY23 | 37000±2000 | 1.1 | -26.5 | 20.5 |
| AMOT PY34 | YAP WW12 | 69000±8200 | 1.5 | -15 | 9.3 |
| YAP WW12 | AMOT PY12 | 860±30 | 0.69 | -43.4 | 35.2 |
| YAP WW12 | AMOT PY1-4 | 800±100 | 0.74 | -48.4 | 40 |
| YAP WW12 | AMOT PY1 | 1500±100 | 0.8 | -31.7 | 23.7 |
| YAP WW12 | AMOT PY2 | 6300±200 | 0.46 | -41.6 | 34.6 |
| YAP WW12 | LATS1 PY23 | 3400±200 | 0.65 | -29.7 | 22.2 |
| YAP WW12 | LATS1 PY1-4 | 2500±200 | 1.0 | -34.9 | 27.3 |
| YAP WW12 | LATS1 PY1-3 | 1500±100 | 0.92 | -37 | 29.1 |
| YAP WW12 | LATS1 PY1 | 8500±200 | 0.49 | -38.8 | 31.9 |
| YAP WW12 | Expanded PY12 | 380±10 | 0.74 | -40.9 | 32.2 |
| PTPN14 PY12 | KIBRA WW12 I35D | 410±20 | 0.74 | -51.8 | 43.2 |
| PTPN14 PY12 | KIBRA WW12 F47A | 88±5 | 0.77 | -51.2 | 41.4 |
| PTPN14 PY12 | KIBRA WW12 L57D | 277±13 | 0.74 | -48.6 | 39.7 |
| PTPN14 PY12 | KIBRA WW12 W88A | 15±1 | 0.77 | -54.2 | 43.5 |
| PTPN14 PY12 | KIBRA 5-87 | 63±6 | 0.78 | -55 | 45 |
| PTPN14 PY12 | MAGI2 WW12 | 55±7 | 0.99 | -30.6 | 20 |
| PTPN14 PY12 | MAGI3 WW12 | 103±2 | 0.96 | -33.9 | 24.4 |
| PTPN14 PY12 | MAGI1 WW12 | 3980±270 | 0.97 | -24.1 | 16.8 |
| PTPN14 PY12 | SAV1 WW12 | n.d. |  |  |  |
| PTPN14 PY12 | WWOX WW12 | n.d. |  |  |  |
| PTPN14 PY12 | ITCH WW12 | 20400±3600 | 0.78 | -10 | 3.58 |
| PTPN14 PY12 | ITCH WW34 | 63700±8700 | n.r. | n.r. | n.r. |
| LATS1 PY23 | MAGI2 WW12 | 650±20 | 0.98 | -26.6 | 18.2 |
| LATS1 PY23 | MAGI3 WW12 | 470±10 | 0.97 | -29.5 | 20.8 |
| LATS1 PY23 | MAGI1 WW12 | 19000±1000 | 1.2 | -20.9 | 14.5 |
| LATS1 PY23 | SAV1 WW12 | n.d. |  |  |  |
| LATS1 PY23 | WWOX WW12 | n.d. |  |  |  |
| LATS1 PY23 | ITCH WW12 | 25000±5000 | 1.1 | -19.9 | 13.6 |
| LATS1 PY23 | ITCH WW34 | 34000±3000 | 1.2 | -15 | 8.9 |
| Dendrin PY23 | MAGI1 WW12 | 60±3 | 0.83 | -34.3 | 24.4 |
| Dendrin PY23 | MAGI2 WW12 | 2.3±0.2 | 0.84 | -37.8 | 26 |
| Dendrin PY23 | MAGI3 WW12 | 3.6±0.9 | 0.88 | -39 | 27.5 |
| AMOT PY34 | MAGI3 WW12 | 460±20 | 1.1 | -25.1 | 16.5 |
| Expanded PY12 | MAGI1 WW12 | 2900±200 | 1.1 | -34.7 | 27.2 |
| Expanded PY12 | MAIG3 WW12 | 3300±150 | 0.67 | -34.4 | 26.9 |
| Yorkie WW12 | Dendrin PY23 | 500±20 | 0.82 | -37.6 | 29 |
| Dendrin PY23 | MAGI2 WW12 L330D | 130±10 | 0.94 | -32.4 | 23 |
| Dendrin PY23 | YAP WW12 L244D | 310±10 | 0.83 | -37.9 | 29 |
| YAP WW12 L244D | AMOT PY12 | 740±20 | 0.79 | -42.8 | 34.3 |
| PTPN14 PY12 1res | KIBRA WW12 | 36000±9000 | 0.75 | -22.2 | 16.2 |
| PTPN14 PY12 1res | MAGI3 WW12 | 12200±1100 | 0.91 | -16.9 | 10.1 |
| PTPN14 PY12 1res | YAP WW12 | 74000±7200 | 0.77 | -25.1 | 2.0 |
| PTPN14 PY12 3res | KIBRA WW12 | 3500±150 | 0.83 | -40.1 | 32.8 |
| PTPN14 PY12 3res | MAGI3 WW12 | 1000±30 | 0.94 | -24.7 | 16.5 |
| PTPN14 PY12 3res | YAP WW12 | 18400±370 | 1.0 | -25.2 | 18.7 |
| AMOT PY12 | MAGI3 WW12 | 1630±100 | 0.9 | -38.1 | 30.4 |
| LATS1 PY1-4 G553E | KIBRA WW12 | 4000±100 | 0.65 | -52.4 | 45 |
| PTPN14 PY12 R440A | KIBRA WW12 | 140±10 | 0.74 | -53.6 | 44.1 |
| PTPN14 PY12 Del-C | KIBRA WW12 | 180±10 | 0.79 | -51.5 | 42.3 |
| KIBRA WW12 | PTPN14 PY12 2Pro:YAP1=1:2 | 0.9±0.07 | 1.1 | -50.3 | 38 |
| KIBRA WW12 | PTPN14 PY12 4Pro:YAP1=1:2 | 0.5±0.06 | 1.0 | -50.7 | 38 |
| PTPN14 PY12 R440A | MAGI3 WW12 | 360±10 | 0.92 | -34.4 | 25.6 |
| PTPN14 PY12 Del-C | MAGI3 WW12 | 7100±300 | 0.88 | -32.2 | 25.2 |
| PTPN14 PY12 2Pro | MAGI3 WW12 | 4.0±0.3 | 0.93 | -31.8 | 20.4 |
| PTPN14 PY12 4Pro | MAGI3 WW12 | 1.0±0.2 | 0.99 | -25.8 | 13.5 |
| DRPPPYVAPPSYEG | KIBRA WW12 | 62±3 | 0.91 | -48.8 | 39.1 |
| DRPPPYVACPSYEG | KIBRA WW12 | 200±10 | 0.93 | -50.3 | 41.1 |
| DRPPPYVAAPSYEG | KIBRA WW12 | 230±10 | 0.88 | -52.1 | 42.9 |
| DRPPPYVASPSYEG | KIBRA WW12 | 390±10 | 0.89 | -51.2 | 42.6 |
| DRPPPYVATPSYEG | KIBRA WW12 | 980±20 | 0.92 | -48.8 | 40.5 |
| DRPPPYVAVPSYEG | KIBRA WW12 | 1100±100 | 0.95 | -47.9 | 39.7 |
| DRPPPYVALPSYEG | KIBRA WW12 | 23000±2000 | n.r. | n.r. | n.r. |
| DRPPPYVAMPSYEG | KIBRA WW12 | 22000±2000 | n.r. | n.r. | n.r. |
| DRPPPYVAIPSYEG | KIBRA WW12 | 23000±3000 | n.r. | n.r. | n.r. |
| DRPPPYVAPPSYEG | MAGI3 WW12 | 18±1 | 0.98 | -32 | 21.4 |
| DRPPPYVACPSYEG | MAGI3 WW12 | 150±10 | 1.0 | -32.7 | 23.3 |
| DRPPPYVAAPSYEG | MAGI3 WW12 | 100±10 | 0.96 | -33.6 | 24.1 |
| DRPPPYVASPSYEG | MAGI3 WW12 | 280±10 | 0.99 | -33.7 | 24.7 |
| DRPPPYVATPSYEG | MAGI3 WW12 | 650±10 | 0.99 | -31.8 | 23.3 |
| DRPPPYVAVPSYEG | MAGI3 WW12 | 1400±20 | 1.0 | -32.1 | 24.1 |
| DRPPPYVALPSYEG | MAGI3 WW12 | 6200±600 | 1.7 | -13.4 | 6.3 |
| DRPPPYVAMPSYEG | MAGI3 WW12 | 4600±400 | 1.6 | -13.2 | 5.9 |
| DRPPPYVAIPSYEG | MAGI3 WW12 | 6200±1100 | 1.9 | -11.2 | 4.1 |
| KIBRA WW12 | JCAD PY23 | 58±3 | 1.0 | -51.3 | 41.4 |
| USP6NL PY12 | KIBRA WW12 | 110±10 | 0.9 | -48.1 | 38.5 |
| β-Dystroglycan PY34 | KIBRA WW12 | 96±8 | 1.2 | -34.9 | 25.3 |
| KIBRA WW12 | PTPN21 PY12:YAP1=1:2 | 8±0.5 | 1.2 | -52.7 | 41.6 |
| PTPN21 PY34 | KIBRA WW12 | 135±9 | 0.82 | -39.5 | 30.1 |
| ABLIM1 PY12 | KIBRA WW12 | 530±10 | 1.0 | -35.8 | 27.2 |
| PTCH1 PY12 | KIBRA WW12 | 1100±30 | 0.9 | -39.6 | 31.6 |
| MAGI3 WW12 | JCAD PY23 | 22±1 | 0.97 | -36.3 | 25.8 |
| USP6NL PY12 | MAGI3 WW12 | 200±10 | 0.93 | -30.8 | 21.6 |
| β-Dystroglycan PY34 | MAGI3 WW12 | 2700±50 | 1.1 | -20.7 | 13.1 |
| PTPN21 PY12 | MAGI3 WW12 | 49±1 | 0.96 | -33.3 | 23.4 |
| PTPN21 PY34 | MAGI3 WW12 | 780±70 | 1.1 | -22 | 13.6 |
| ABLIM1 PY12 | MAGI3 WW12 | 2100±40 | 1.1 | -20.2 | 12.5 |
| PTCH1 PY12 | MAGI3 WW12 | 900±20 | 0.94 | -23.7 | 15.4 |

n.r. stands for not reliable due to poor curve fitting; n.d. stands for non-detectable
