# Supplementary material for: Decoding WW domain tandem-mediated target recognitions in tissue growth and cell polarity
Source: eLife. 2019 Sep 5;8:e49439. doi: 10.7554/eLife.49439 (PMC6744271; doi:10.7554/eLife.49439)
Supplement: Supplementary file 3. [file elife-49439-supp3.docx]

**Supplementary file 3: Human proteins containing the “ΦΦxYxxΨPxY” motif**

| **UniProt Name** | **Sequence** |
| --- | --- |
| AAAD_HUMAN | TADRLDAVVV**STNYRLAPKY**HFPIQFEDVY |
| ABL1_HUMAN | LHyPAPKRNK**PtVyGVsPNy**DKWEMERTDI |
| ABLM1_HUMAN | PKHFHVPDQG**INIyRKPPIy**KQHAALAAQs |
| ABLM3_HUMAN^1^ | NLDLRQRRAs**SPGYIDsPTY**sRQGMsPtFS |
| ABLM3_HUMAN^2^ | PKHFHIPAGD**SNIYRKPPIY**KRHGDLSTAT |
| AG10A_HUMAN | VWKRVFQRyE**tVKYLLVPAY**IFAGWSIADs |
| AG10B_HUMAN | VWKRVFQRYA**ILKYLLVPAY**IFAGWSIADS |
| AGRB1_HUMAN | ILRRCELDEE**GIAYWEPPTY**IRCVSIDYRN |
| AMOL1_HUMAN | APQPVRTDVA**VLRYQPPPEY**GVtsRPCQLP |
| AMOT_HUMAN | LNQPGRTEGQ**LMRYQHPPEY**GAARPAQDIS |
| ANKL2_HUMAN | RAGAtAsKEP**PLyyGVCPVy**EDVPARNERI |
| ARI1B_HUMAN | YGPQMSQYGP**QGNYSRPPAY**SGVPSASYSG |
| ARRD1_HUMAN | LLIPPEySSW**GYPYEAPPSY**EQSCGGVEPS |
| AT7L1_HUMAN | KNCVLNASSA**LNSYQAAPPY**NSLSVVHKKN |
| B3GA2_HUMAN | RPQPQPEPQL**PTIYAITPTY**SRPVQKAELT |
| B3GA3_HUMAN | PAQPPEPEAL**PTIYVVTPTY**ARLVQKAELV |
| BAT1_HUMAN | PTSFAIICLS**FSEYVCAPFY**VGCKPPQIVV |
| BGH3_HUMAN | YQRKICGKST**VISYECCPGY**EKVPGEKGCP |
| BGP11_HUMAN | FFSYAGLLWS**LLLYTTPPTY**IDDVTVTTGV |
| CA054_HUMAN | RLGEDEYYQV**VYYYTVTPSY**DDFSADFTID |
| CALCR_HUMAN | WLCWDDTPAG**VLSYQFCPDY**FPDFDPSEKV |
| CASP3_HUMAN | CHKIPVEADF**LYAYSTAPGY**YSWRNSKDGS |
| CASP7_HUMAN | RYKIPVEADF**LFAYSTVPGY**YSWRSPGRGs |
| CC130_HUMAN | GVRYNAEKKK**VGNYYTTPIY**RFRMKCHLCV |
| CD166_HUMAN | FIAFRSSTKK**SVQyDDVPEy**KDRLNLSENY |
| CD1B_HUMAN | QYQGIMETVR**ILLYETCPRY**LLGVLNAGKA |
| CENPL_HUMAN | QKVAFLLHKQ**WTLYSLTPLY**KFSYSNLKEY |
| CHD3_HUMAN | TEPHPTPAYP**PGPYATPPGY**GAAFSAAPVG |
| CI114_HUMAN | KGQACVQLAR**ILQYLECPQY**LRKAFFPKHQ |
| CK053_HUMAN | SLHALEDLHH**TPGYPTPPPY**PFTPFMTVSN |
| COX11_HUMAN | YVAAVAVGML**GASYAAVPLy**RLYCQTTGLG |
| CP089_HUMAN | KQFSCLILPS**SWDYRSVPPY**LANFYIFLVE |
| CRIP3_HUMAN | LTAGSHAEHD**GVPYCHVPCY**GYLFGPKGGQ |
| CRLF3_HUMAN | TSNNEGGHFS**GVLYSRAPTY**FCGQTLTFRV |
| CSMD2_HUMAN | RLGNDFRYNK**TVTYQCVPGY**MMESHRVSVL |
| CSMD3_HUMAN | LEGTNFDWGF**SISYICSPGY**ELSFPAVLTC |
| CUBN_HUMAN | VSRCGSNFTG**PSGYIISPNY**PKQYDNNMNC |
| CYYR1_HUMAN | GILRTTHINT**VSSYPGPPPY**GHDHEMEYCA |
| DAG1_HUMAN | MEGKGSRPKN**MtPyRsPPPy**VPP |
| DEMA_HUMAN | FHHPETSRPD**sNIyKKPPIY**KQREsVGGsP |
| DEND_HUMAN | GTAPRRRWDR**PPPYVAPPSY**EGPHRTLGTK |
| DHX33_HUMAN | YMRDLCVIDA**QWLYEAAPEY**FRRKLRTARN |
| DLX3_HUMAN | PAPARSQLPP**PLPYSASPSY**LDDPTNSWYH |
| DOT1L_HUMAN | LESFKIQYLQ**FLAYTKTPQY**KASLQELLGQ |
| DTHD1_HUMAN | DVLSDVTGPQ**VSCYITAPSY**VLQQLECRII |
| EGR2_HUMAN | LSAATTSTSS**SLAYPPPPSY**PSPKPATDPG |
| ELFN1_HUMAN | AySQLSPQyH**SLSYSSSPEY**TCRASQSIWE |
| FOXL2_HUMAN | GCGVAGAGAD**GYGYLAPPKY**LQSGFLNNSW |
| FR1L5_HUMAN | STPYSPVIYD**GNIYHYVPWY**NTKPVVAVTS |
| GCM1_HUMAN | NLYEEKVHVD**FNSYVQSPAY**HSPQEDPFLF |
| GGH_HUMAN | HFKSESEEEK**ALIYQFSPIY**TGNISSFQQC |
| GRK5_HUMAN | VGYMAPEVLN**NQRYGLSPDY**WGLGCLIYEM |
| GT251_HUMAN | YSNFWCGMTS**QGYYKRTPAy**IPIRKRDRRG |
| HDAC8_HUMAN | EEPADSGQSL**VPVYIYSPEY**VSMCDSLAKI |
| HXC10_HUMAN | SCLGEHEVPV**PSYYRAsPsY**sALDKtPHCS |
| HXD13_HUMAN | AKEVSFYQGY**TSPYQHVPGY**IDMVSTFGSG |
| IRX1_HUMAN | VTSVLGMYAA**AGPYAGAPNY**SAFLPYAADL |
| JCAD_HUMAN | ELCLSDPGLE**PPVyVPPPSY**RSPPQNIPNP |
| JKAMP_HUMAN | CRVLMLSDWY**TMLYNPSPDY**VTTVHCTHEA |
| KDIS_HUMAN | PQHPFYNRPF**FAPYLyTPRy**yPGGsQHLIS |
| KLK5_HUMAN | HCRKKVFRVR**LGHYSLSPVY**ESGQQMFQGV |
| KRA61_HUMAN | MCGSY**YGNYYGTPGY**GFCGYGGLGY |
| LAPM5_HUMAN | LDFCLSILTL**CSSYMEVPTY**LNFKSMNHMN |
| LCTL_HUMAN | WGFRRLLNFA**QTQYGDPPIY**VMENGASQKF |
| LMBD1_HUMAN | ILLLIVLHTS**YMIYSLAPQY**VMYGSQNYLI |
| LRC15_HUMAN | VAVPSVHVPE**VPSYPETPWY**PDTPSYPDTT |
| MBOA1_HUMAN | WNIQTATWLK**CVCYQRVPWY**PTVLTFILSA |
| MD12L_HUMAN | SSSRVDEYPQ**SNIYRVPPNY**SPISSQMMHH |
| MPIP1_HUMAN | KGGYKEFFMK**CQSYCEPPSY**RPMHHEDFKE |
| MTMR4_HUMAN | LQNVWRVSHI**NSNYKLCPSY**PQKLLVPVWI |
| MYO9A_HUMAN | TASSHGTRKL**FQIySKSPFY**RAAsGNEALG |
| NEBU_HUMAN | KENyEKTKAK**SMNYCETPKY**QLDTQLKNFS |
| NEK3_HUMAN | ARLLSNPMAF**ACTYVGTPYY**VPPEIWENLP |
| NFAC4_HUMAN | PACETPYLSE**GFGYGMPPLY**PQTGPPPSYR |
| NOS2_HUMAN | GSITPVFHQE**MLNYVLSPFY**YYQVEAWKTH |
| NRP1_HUMAN | DKCGDtIKIE**SPGYLTSPGY**PHsYHPsEKC |
| PCSK6_HUMAN | YDSYASYDVN**GNDYDPSPRY**DASNENKHGT |
| PLS3_HUMAN | LPPKGYAPSP**PPPYPVtPGY**PEPALHPGPG |
| PORED_HUMAN | HCNHRIPFGD**WFEYVSSPNY**LAELMIYVSM |
| POSTN_HUMAN | YKKSICGQKT**TVLYECCPGy**MRMEGMKGCP |
| PPR29_HUMAN | TySQLsPRHy**YSGySssPEy**SSESTHKIWE |
| PRRX2_HUMAN | LSPDYLSWTA**SSPYSTVPPY**SPGSSGPATP |
| PTC1_HUMAN | EPQAYTDTHD**NTRYSPPPPY**SSHSFAHETQ |
| PTN14_HUMAN | IPSHRHsAII**VPSyRPTPDy**EtVMRQMKRG |
| PTN21_HUMAN^1^ | LPSHRHSAVI**PPSYRPTPDY**ETVMKQLNRG |
| PTN21_HUMAN^2^ | QDYPSPNIMR**TQVYRPPPPy**PPPRPANstP |
| PTPRQ_HUMAN | INYKNISSSS**ILLYWDPPEY**PNGKITHYTI |
| R3HD1_HUMAN | NSIGNQIQGV**VIPYTSVPTY**QVSLPQGSQG |
| RBP2_HUMAN | APLTVATTGP**SVYYSQSPAY**NSQYLLRPAA |
| RFX1_HUMAN | SYTASAIRSS**TYSYPETPLY**TQTASTSYYE |
| RGPD1_HUMAN | APLTVATTGP**SVYYSQSPAY**NSQYLLRPAA |
| RGPD2_HUMAN | APLTVATTGP**SVYYSQSPAY**NSQYLLRPAA |
| RGPD3_HUMAN | APLTVATTGP**SVYYSQSPAY**NSQYLLRPAA |
| RGPD4_HUMAN | APLTVATTGP**SVYYSQSPAY**NSQYLLRPAA |
| RGPD5_HUMAN | APLTVATTGP**SVYYSQSPAY**NSQYLLRPAA |
| RGPD8_HUMAN | APLTVATTGP**SVYYSQSPAY**NSQYLLRPAA |
| RGS9_HUMAN | HIYMLMKKDS**YARYLKSPIY**KDMLAKAIEP |
| RN208_HUMAN | CLHSVCEQCL**QILYESCPKy**KFISCPTCRR |
| RNC_HUMAN | HPPPPPVMPQ**QVNYQYPPGY**SHHNFPPPSF |
| ROR1_HUMAN | PIPQNQRFIP**INGYPIPPGy**AAFPAAHyQP |
| ROR2_HUMAN | MVPPPQLYVP**VNGyQPVPAY**GAYLPNFYPV |
| RT15_HUMAN | DVFEKICWGL**GIEyTFPPLY**YRRAHRRFVT |
| S14L4_HUMAN | IQLYDSGGLC**GYDYEGCPVY**FNIIGSLDPK |
| S2535_HUMAN | SAAAGAMAGV**MGAYLGSPIY**MVKTHLQAQA |
| SAXO1_HUMAN | SFTPKEMGRC**LASYPEPPGY**TFEEVDALGH |
| SAXO2_HUMAN | EyTPKRQEIC**PASYPSPPGY**IFDNTNSQGH |
| SC16B_HUMAN | ENTFYQDFSG**CQGYSEAPGY**RSALWLTPEQ |
| SC24D_HUMAN | MS**QQGYVATPPY**SQPQPGIGLS |
| SCAM4_HUMAN | AQyNNFSGNS**LPEYPTVPSy**PGSGQWP |
| SCAM5_HUMAN | GAAQGAMNQP**QTQYSATPNY**TYSNEM |
| SCUB1_HUMAN | NQHCGGELGD**YTGYIESPNY**PGDYPANAEC |
| SCUB2_HUMAN | NRRCGGELGD**FTGYIESPNY**PGNYPANTEC |
| SCUB3_HUMAN | NRQCGGELGE**FTGYIESPNY**PGNYPAGVEC |
| SF3B2_HUMAN | HKVPPPWLIA**MQRYGPPPSY**PNLKIPGLNs |
| SH321_HUMAN | PGPQRPPKLS**SLAYDSPPDY**LQTVSHPEVY |
| SIAE_HUMAN | VALRYAWTTW**PCEYKQCPLY**HPSSALPAPP |
| SIDT1_HUMAN | GIALMMEGVL**SACYHVCPNY**SNFQFDTSFM |
| SIDT2_HUMAN | GTALMMEGLL**SACYHVCPNY**TNFQFDTSFM |
| SIN3A_HUMAN | AVSETMQSAT**GIQYSVTPSY**QVSAMPQSSG |
| SLIK5_HUMAN | LPEYPKFPCS**PAAYTFSPNy**DLRRPHQYLH |
| SO5A1_HUMAN | ALGMQFVLLR**TLAYIPTPIY**FGAVIDTTCM |
| SPF27_HUMAN | ETRRYRPTKN**YLSYLTAPDY**SAFETDIMRN |
| SPT5H_HUMAN | TVGSRRPGGM**tStyGRtPMy**GsQtPMyGsG |
| STK31_HUMAN | LIFLFLCKSD**PMAYLMVPYY**PRANLNAVQA |
| TASOR_HUMAN | PLGDRGyLFL**LSPyQMVPPY**EyQTAKSRVL |
| TPTE2_HUMAN | LHDIETDKIL**INVYDGPPLY**DDVKVQFFSS |
| TRYG1_HUMAN | WGEGCGRPNR**PGVYTRVPAY**VNWIRRHITA |
| TS1R2_HUMAN | PMCFLMLTLL**LVAYMVVPVY**VGPPKVSTCL |
| US6NL_HUMAN | LDGEARGLAH**PPsysNPPVy**HGNsPKHFPt |
| WBP2_HUMAN | GAYVYPPPVA**NGMYPCPPGY**PYPPPPPEFY |
| ZN462_HUMAN | SGQNATSLGT**GGYYGHSPGY**YGQHIAANPK |
| ZN526_HUMAN | AFARAPRLPI**TGLYNKSPYY**CGTCGRWFRA |
| ZN573_HUMAN | QFTDLDLQCE**IISYIEVPTY**ETDISSTQLQ |
| ZN609_HUMAN | RPNMYMQSLY**YNQYAYVPPY**GYSDQSYHTH |
