# Supplementary material for: Decoding WW domain tandem-mediated target recognitions in tissue growth and cell polarity
Source: eLife. 2019 Sep 5;8:e49439. doi: 10.7554/eLife.49439 (PMC6744271; doi:10.7554/eLife.49439)
Supplement: Supplementary file 4. [file elife-49439-supp4.docx]

**Supplementary file 4: Key Resources Table**

| **Reagent type (species) or resource** | **Designation** | **Source or reference** | **Identifiers** | **Additional information** |
| --- | --- | --- | --- | --- |
| Strain, strain background (*E. coli*) | BL21(DE3) | Novagen | Cat# 69450 |  |
| Strain, strain background (*E. coli*) | B834(DE3) | Novagen | Cat# 69041 |  |
| Cell line (*Homo-sapiens*) | HEK293A | ATCC | RRID: CVCL_6910 |  |
| Cell line (*Homo-sapiens*) | HEK293A-LATS1/2 dKO | PMID: 26437443 | NCBI: NM_004690.4; NM_014572.3 | Immunoblotting Figure 5 |
| Cell line (*Homo-sapiens*) | HEK293A- LATS1/2 dKO-LATS1-WT | This paper | NCBI: NM_004690.4; NM_014572.3 | Immunoblotting Figure 5 |
| Cell line (*Homo-sapiens*) | HEK293A- LATS1/2 dKO-LATS1-△553; delete G554 | This paper | NCBI: NM_004690.4; NM_014572.3 | Immunoblotting Figure 5 |
| Peptide, recombinant protein | KIBRA and PTPN14 complex: mouse KIBRA aa E5-H132; human PTPN14 PY12 aa A433-G455 | This paper | UniProt: Q5SXA9; Q15678 | Crystallization |
| Peptide, recombinant protein | KIBRA and AMOT complex: mouse KIBRA aa E5-H132; human AMOT PY34 aa G272-A293 | This paper | UniProt: Q5SXA9; Q4VCS5 | Crystallization |
| Peptide, recombinant protein | KIBRA and LATS1 complex: mouse KIBRA aa E5-H132; mouse LATS1 PY23 aa V545-S568 | This paper | UniProt: Q5SXA9; Q8BYR2 | Crystallization |
| Peptide, recombinant protein | KIBRA andβ-Dystroglycan complex: mouse KIBRA aa E5-H132; humanβ-Dystroglycan PY34 aa A433-G455 | This paper | UniProt: Q5SXA9; Q14118 | Crystallization |
| Peptide, recombinant protein | MAGI2 and Dendrin complex: mouse MAGI2 aa E295-N390; mouse Dendrin aa D222-G241 | This paper | UniProt: Q9WVQ1; Q80TS7 | Crystallization |
| Peptide, recombinant protein | Dendrin-“L”-YAP fusion: mouse Dendrin aa D222-G241; mouse YAP aa V156-R247 | This paper | UniProt: Q80TS7; P46938 | Crystallization |
| Peptide, recombinant protein | His_6_-YAP-“L”-Dendrin fusion: mouse YAP aa V156-R247; mouse Dendrin aa D222-G241 | This paper | UniProt: P46938; Q80TS7 | Crystallization |
| Peptide, recombinant protein | His_6_-KIBRA Mouse WW12 WT: aa E5-K97 | This paper | UniProt: Q5SXA9 | ITC, Figure 1, 3, 4, 6, 7; Gel filtration, Figure 5 |
| Peptide, recombinant protein | His_6_-KIBRA Mouse WW12 I35D | This paper | UniProt: Q5SXA9 | ITC, Figure 3, 4 |
| Peptide, recombinant protein | His_6_-KIBRA Mouse WW12 F47A | This paper | UniProt:Q5SXA9 | ITC, Figure 3 |
| Peptide, recombinant protein | His_6_-KIBRA Mouse WW12 L57D | This paper | UniProt: Q5SXA9 | ITC, Figure 3 |
| Peptide, recombinant protein | His_6_-KIBRA Mouse WW12 W88A | This paper | UniProt: Q5SXA9 | ITC, Figure 3 |
| Peptide, recombinant protein | His_6_-KIBRA Mouse WT: aa E5-Q87 | This paper | UniProt: Q5SXA9 | ITC, Figure 3 |
| Peptide, recombinant protein | His_6_-KIBRA Mouse WW1 WT: aa E5-K43 | This paper | UniProt: Q5SXA9 | ITC, Figure 1, 4 |
| Peptide, recombinant protein | TRX-KIBRA Mouse WW1 WT： aa K43-K97 | This paper | UniProt: Q5SXA9 | ITC, Figure 1, 4 |
| Peptide, recombinant protein | TRX-YAP Mouse WW12 WT：aa E151-T259 | This paper | UniProt: P46938 | ITC, Figure 1, 3, 4 |
| Peptide, recombinant protein | TRX-YAP Mouse WW12 L224D | This paper | UniProt: P46938 | ITC, Figure 4 |
| Peptide, recombinant protein | TRX-YAP Mouse WW1 WT: aa E151-P216 | This paper | UniProt: P46938 | ITC, Figure 1, 4 |
| Peptide, recombinant protein | TRX-YAP Mouse WW2 WT: aa R188-T259 | This paper | UniProt: P46938 | ITC, Figure 1, 4 |
| Peptide, recombinant protein | TRX-MAGI1 Mouse WW12 WT: aa A295-Q402 | This paper | UniProt: Q6RHR9 | ITC, Figure 3 |
| Peptide, recombinant protein | His_6_-MAGI2 Mouse WW12 WT: aa E295-N390 | This paper | UniProt: Q9WVQ1 | ITC, Figure 3, 4 |
| Peptide, recombinant protein | His_6_-MAGI2 Mouse WW12; L330D | This paper | UniProt: Q9WVQ1 | ITC, Figure 4 |
| Peptide, recombinant protein | His_6_-MAGI3 Rat WW12 WT: aa R291-G384 | This paper | UniProt: Q9JK71 | ITC, Figure 3, 4, 6, 7 |
| Peptide, recombinant protein | TRX-SAV1 Mouse WW12 WT: aa G196-A268 | This paper | UniProt: Q8VEB2 | ITC, Figure 3 |
| Peptide, recombinant protein | His_6_-WWOX Mouse WW12 WT: aa M1-Y107 | This paper | UniProt: Q91WL8 | ITC, Figure 3 |
| Peptide, recombinant protein | TRX-ITCH Mouse WW12 WT: aa A287-L352 | This paper | UniProt: Q8C863 | ITC, Figure 3 |
| Peptide, recombinant protein | TRX-ITCH Mouse WW34 WT: aa G399-T472 | This paper | UniProt: Q8C863 | ITC, Figure 3 |
| Peptide, recombinant protein | His_6_-Yorkie Drosophila WW12 WT: aa G241-M343 | This paper | UniProt: Q45VV3 | ITC, Figure 4 |
| Peptide, recombinant protein | TRX-PTPN14 Human PY12 WT: aa A433-G455 | This paper | UniProt: Q15678 | ITC, Figure 1, 3, 4, 6 |
| Peptide, recombinant protein | TRX-PTPN14 Human PY12-Del-C WT: aa A433-T447 | This paper | UniProt: Q15678 | ITC, Figure 6 |
| Peptide, recombinant protein | TRX-PTPN14 Human PY12; R440A | This paper | UniProt: Q15678 | ITC, Figure 6 |
| Peptide, recombinant protein | TRX-PTPN14 Human PY12 2Pro; V436P and T442P | This paper | UniProt: Q15678 | ITC, Figure 6 |
| Peptide, recombinant protein | TRX-PTPN14 Human PY12 4Pro; V436P and S438P and T442P and D444P | This paper | UniProt: Q15678 | ITC, Figure 6 |
| Peptide, recombinant protein | TRX-Dendrin Mouse PY23 WT: aa A216-R250 | This paper | UniProt: Q80TS7 | ITC, Figure 1, 3, 4 |
| Peptide, recombinant protein | TRX-AMOT Human PY1-4 WT: aa M1-A293 | This paper | UniProt: Q4VCS5 | ITC, Figure 1 |
| Peptide, recombinant protein | TRX-AMOT Human PY12 WT: aa M1-Q270 | This paper | UniProt: Q4VCS5 | ITC, Figure 1, 4 |
| Peptide, recombinant protein | TRX-AMOT Human PY34 WT: aa G272-A293 | This paper | UniProt: Q4VCS5 | ITC, Figure 1, 3 |
| Peptide, recombinant protein | TRX-AMOT Human PY1 WT: aa M1-P201 | This paper | UniProt: Q4VCS5 | ITC, Figure 1 |
| Peptide, recombinant protein | TRX-AMOT Human PY2 WT: aa K231-Q270 | This paper | UniProt: Q4VCS5 | ITC, Figure 1 |
| Peptide, recombinant protein | TRX-LATS1 Mouse PY1-4 WT: aa N361-E582 | This paper | UniProt: Q8BYR2 | ITC, Figure 1, 5; Gel filtration, Figure 5 |
| Peptide, recombinant protein | TRX-LATS1 Mouse PY1-4 G553E | This paper | UniProt: Q8BYR2 | ITC, Figure 5; Gel filtration, Figure 5 |
| Peptide, recombinant protein | TRX-LATS1 Mouse PY1-4-Δ553; delete G553 | This paper | UniProt: Q8BYR2 | Gel filtration, Figure 5 |
| Peptide, recombinant protein | MBP-YAP Mouse WW12 WT：aa E151-T259 | This paper | UniProt: P46938 | Gel filtration, Figure 5 |
| Peptide, recombinant protein | TRX-LATS1 Mouse PY1-3 WT: aa N361-S568 | This paper | UniProt: Q8BYR2 | ITC, Figure 1 |
| Peptide, recombinant protein | TRX-LATS1 Mouse PY1 WT: aa N361-G534 | This paper | UniProt: Q8BYR2 | ITC, Figure 1 |
| Peptide, recombinant protein | TRX-LATS1 Mouse PY23 WT aa V545-S568 | This paper | UniProt: Q8BYR2 | ITC, Figure 1, 3 |
| Peptide, recombinant protein | TRX-LATS1 Mouse PY23-Δ553; delete G553 | This paper | UniProt: Q8BYR2 | ITC, Figure 4 |
| Peptide, recombinant protein | TRX-Expanded Drosophila PY12 WT: aa G748-T856 | This paper | UniProt: Q07436 | ITC, Figure 1, 4 |
| Peptide, recombinant protein | TRX-JCAD Human PY23 WT: aa D332-P354 | This paper | UniProt: Q9P266 | ITC, Figure 7 |
| Peptide, recombinant protein | TRX-USP6NL Human PY12 WT: aa R624-T648 | This paper | UniProt: Q92738 | ITC, Figure 7 |
| Peptide, recombinant protein | TRX-β-Dystroglycan Human PY34 WT: aa R879-P895 | This paper | UniProt: Q14118 | ITC, Figure 7 |
| Peptide, recombinant protein | TRX-PTPN21 Human PY12 WT: aa A439-G461 | This paper | UniProt: Q16825 | ITC, Figure 7 |
| Peptide, recombinant protein | TRX-PTPN21 Human PY34 WT: aa P555-N576 | This paper | UniProt: Q16825 | ITC, Figure 7 |
| Peptide, recombinant protein | TRX-ABLIM1 Human PY12 WT: aa G518-Q537 | This paper | UniProt: O14639 | ITC, Figure 7 |
| Peptide, recombinant protein | TRX-PTCH1 Human PY12 aa H636-T657 | This paper | UniProt: Q13635 | ITC, Figure 7 |
| Peptide, recombinant protein | TRX-Peptide (DRPPPYVAPPSYEG) | This paper | N/A | ITC, Figure 6 |
| Peptide, recombinant protein | TRX-Peptide (DRPPPYVACPSYEG) | This paper | N/A | ITC, Figure 6 |
| Peptide, recombinant protein | TRX-Peptide (DRPPPYVAAPSYEG) | This paper | N/A | ITC, Figure 6 |
| Peptide, recombinant protein | TRX-Peptide (DRPPPYVASPSYEG) | This paper | N/A | ITC, Figure 6 |
| Peptide, recombinant protein | TRX-Peptide (DRPPPYVATPSYEG) | This paper | N/A | ITC, Figure 6 |
| Peptide, recombinant protein | TRX-Peptide (DRPPPYVAVPSYEG) | This paper | N/A | ITC, Figure 6 |
| Peptide, recombinant protein | TRX-Peptide (DRPPPYVALPSYEG) | This paper | N/A | ITC, Figure 6 |
| Peptide, recombinant protein | TRX-Peptide (DRPPPYVAMPSYEG) | This paper | N/A | ITC, Figure 6 |
| Peptide, recombinant protein | TRX-Peptide (DRPPPYVAIPSYEG) | This paper | N/A | ITC, Figure 6 |
| Transfected construct (*Homo-sapiens)* | Flag-KIBRA FL WT: aa M1-V1119 | PMID: 26437443 | NCBI: NM_001161661.2 | Immunoprecipitation, Figure 5 |
| Transfected construct (*Homo-sapiens)* | HA-LATS1 FL WT: aa M1-V1130 | PMID: 26437443 | NCBI: NM_004690.4 | Immunoprecipitation, Figure 5 |
| Transfected construct (*Homo-sapiens)* | HA-LATS1-△553 FL; delete G554 | This paper | NCBI: NM_004690.4 | Immunoprecipitation, Figure 5 |
| Antibody | Anti-Flag (mouse monoclonal) | Sigma-Aldrich | Cat# F1804; RRID:AB_26204 | WB (1:5000), IP (1:500) |
| Antibody | Anti-pLATS1(T1079)  (rabbit monoclonal) | Cell Signaling | Cat# 8654S; RRID:AB_10971635 | WB (1:1000) |
| Antibody | Anti-LATS1 (rabbit monoclonal) | Cell Signaling | Cat# 3477; RRID:AB_2133513 | WB (1:1000) |
| Antibody | Anti-YAP/TAZ (mouse monoclonal) | Santa Cruz | Cat# sc-101199; RRID:AB_1131430 | WB (1:1000) |
| Antibody | Anti-pYAP(S127) (rabbit polyclonal) | Cell Signaling | Cat# 4911S; RRID:AB_2218913 | WB (1:1000) |
| Antibody | Anti-HA-HRP (mouse monoclonal) | Cell Signaling | Cat# 2999S; RRID:AB_1264166 | WB (1:5000) |
| Antibody | Anti-GAPDH (rabbit polyclonal) | Santa Cruz | Cat# sc-25778; RRID:AB_10167668 | WB (1:5000) |
| chemical compound, drug | Latrunculin B (LatB) | Abcam | Cat# ab144291 | 0.2 mg/ml |
| chemical compound, drug | PolyJet | SignaGen Laboratories | Cat# SL100688 |  |
| chemical compound, drug | lysophosphatidic acid (LPA) | Sigma-Aldrich | Cat# L7260-5MG | 1μM |
| chemical compound, drug | cOmplete™, EDTA-free Protease Inhibitor Cocktail | Roche | Cat# 11873580001 |  |
| chemical compound, drug | Pierce™ Protein A/G Magnetic Beads | Thermo Fisher Scientific | Cat# 88803 |  |
| Software, algorithm | Origin7.0 | OriginLab | <http://www.originlab.com/>; RRID: SCR_002815 | ITC titration data analysis |
| Software, algorithm | HKL2000 | HKL Research Inc. | <http://www.hkl-xray.com/> | Diffraction data processing and scaling |
| Software, algorithm | HKL3000 | HKL Research Inc. | <http://www.hkl-xray.com/>; RRID: SCR_015023 | Diffraction data processing and scaling |
| Software, algorithm | PHASER | PMID: 19461840 | <http://www.phenixonline.org/>; RRID: SCR_014219 | Crystal structure determination |
| Software, algorithm | PHENIX | PMID: 20124702 | <http://www.phenixonline.org/>; RRID: SCR_014224 | Model building and refinement |
| Software, algorithm | Coot | PMID: 20383002 | <https://www2.mrc-lmb.cam.ac.uk/personal/pemsley/coot/>; RRID: SCR_014222 | Model building |
| Software, algorithm | MolProbity | PMID: 29067766 | <http://molprobity.manchester.ac.uk>; RRID: SCR_014226 | Model Validation |
| Software, algorithm | PyMOL | DeLano Scientific LLC | <http://www.pymol.org/>; RRID: SCR_000305 | Structure figure plot |
| Software, algorithm | ASTRA6.1 | Wyatt Technology Corporation | <http://www.wyatt.com/products/software/astra.html>; RRID: SCR_016255 | Light-scattering data analysis |
